# Effects of artificial intelligence assistance on endoscopist performance: Comparison of diagnostic performance in superficial esophageal squamous cell carcinoma detection using video‐based models

**DOI:** 10.1002/deo2.70083

**Published:** 2025-05-02

**Authors:** Naoki Aoyama, Keiichiro Nakajo, Maasa Sasabe, Atsushi Inaba, Yuki Nakanishi, Hiroshi Seno, Tomonori Yano

**Affiliations:** ^1^ Department of Gastroenterology and Endoscopy National Cancer Center Hospital East Chiba Japan; ^2^ Department of Gastroenterology and Hepatology Kyoto University Graduate School of Medicine Kyoto Japan; ^3^ NEXT Medical Device Innovation Center National Cancer Center Hospital East Chiba Japan; ^4^ Division of Endoscopy Saitama Cancer Center Saitama Japan

**Keywords:** artificial intelligence, esophageal neoplasms, esophageal squamous cell carcinoma, gastrointestinal endoscopy, narrow band imaging

## Abstract

**Objectives:**

Superficial esophageal squamous cell carcinoma (ESCC) detection is crucial. Although narrow‐band imaging improves detection, its effectiveness is diminished by inexperienced endoscopists. The effects of artificial intelligence (AI) assistance on ESCC detection by endoscopists remain unclear. Therefore, this study aimed to develop and validate an AI model for ESCC detection using endoscopic video analysis and evaluate diagnostic improvements.

**Methods:**

Endoscopic videos with and without ESCC lesions were collected from May 2020 to January 2022. The AI model trained on annotated videos and 18 endoscopists (eight experts, 10 non‐experts) evaluated their diagnostic performance. After 4 weeks, the endoscopists re‐evaluated the test data with AI assistance. Sensitivity, specificity, and accuracy were compared between endoscopists with and without AI assistance.

**Results:**

Training data comprised 280 cases (140 with and 140 without lesions), and test data, 115 cases (52 with and 63 without lesions). In the test data, the median lesion size was 14.5 mm (range: 1–100 mm), with pathological depths ranging from high‐grade intraepithelial to submucosal neoplasia. The model's sensitivity, specificity, and accuracy were 76.0%, 79.4%, and 77.2%, respectively. With AI assistance, endoscopist sensitivity (57.4% vs. 66.5%) and accuracy (68.6% vs. 75.9%) improved significantly, while specificity increased slightly (87.0% vs. 91.6%). Experts demonstrated substantial improvements in sensitivity (59.1% vs. 70.0%) and accuracy (72.1% vs. 79.3%). Non‐expert accuracy increased significantly (65.8% vs. 73.3%), with slight improvements in sensitivity (56.1% vs. 63.7%) and specificity (81.9% vs. 89.2%).

**Conclusions:**

AI assistance enhances ESCC detection and improves endoscopists' diagnostic performance, regardless of experience.

## INTRODUCTION

Esophageal cancer, a significant global health burden, ranks seventh in terms of incidence and sixth in terms of mortality. Esophageal squamous cell carcinoma (ESCC) accounts for >90% of esophageal cancers in certain regions like eastern Asia.^1^ Early ESCC detection is crucial for improving patient outcomes, as the 5‐year survival rate dramatically increases with early diagnosis.^2^, ^3^ However, the accurate and timely diagnosis of superficial ESCC remains challenging, particularly for inexperienced endoscopists.^4^

The narrow‐band imaging technique improves superficial ESCC detection compared with white‐light imaging but requires advanced skills, posing difficulties for less experienced endoscopists.^5^ Identifying subtle endoscopic features demands significant training, and a lack of expertise can result in misdiagnoses or delays.

Artificial intelligence (AI) offers a promising solution for endoscopic diagnosis. AI algorithms, particularly those based on deep learning, have demonstrated remarkable capabilities in image recognition and pattern detection. Several studies have reported successful AI applications in gastrointestinal endoscopic image recognition in various situations, including colorectal polyp and gastric cancer detection.^6^, ^7^, ^8^, ^9^, ^10^, ^11^ While prior studies exist on AI for ESCC diagnosis using video data,^12^, ^13^, ^14^, ^15^, ^16^, ^17^, ^18^, ^19^, ^20^, ^21^ few examine its impact on endoscopists’ diagnostic performance.

This study aimed to develop and validate an AI model for detecting superficial ESCC via video analysis, mimicking real‐world clinical scenarios. The AI model's diagnostic accuracy was compared with that of novice and experienced endoscopists.

## METHODS

### Data collection for AI model development

**FIGURE 1:**
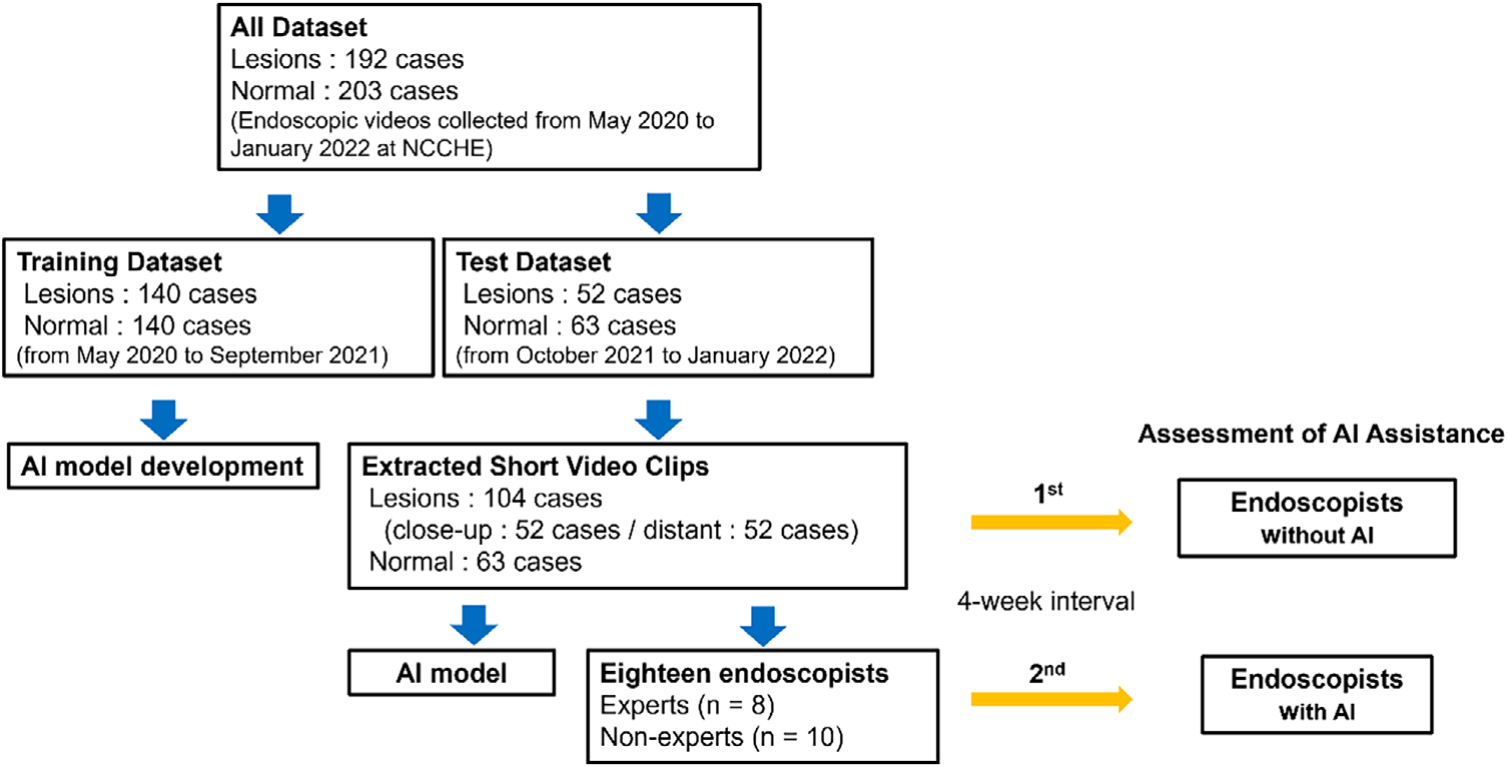
Flowchart detailing the procedures employed in developing and evaluating the AI model for detecting superficial esophageal squamous cell carcinoma, including the assessment of its assistive effects on the performance of endoscopists. AI, artificial intelligence; NCCHE, National Cancer Center Hospital East.

Endoscopic videos were prospectively collected from May 2020 to September 2021 as training data for the AI model. Additional endoscopic videos were collected between October 2021 and January 2022 as test data to evaluate the developed AI model. Videos were obtained during endoscopic procedures at the National Cancer Center Hospital East (NCCHE) using narrow‐band imaging with GIF‐Q260, GIF‐2T260 M, GIF‐H260Z, GIF‐H290Z, GIF‐EZ1500, and GIF‐XZ1200 scopes (Olympus). Additionally, standard video endoscopy systems (EVIS LUCERA CV‐260, EVIS LUCERA ELITE CV‐290, and EVIS X1 CV‐1500; Olympus) were employed. Two types of videos were collected: those with and those without neoplastic lesions. The eligibility criteria for patients with neoplastic lesions included histopathologically diagnosed ESCC or high‐grade intraepithelial neoplasia with lesion depth shallower than the submucosa. Patients with residual or locally recurrent lesions after endoscopic resection were excluded. Patients without neoplastic lesions included those without ESCC or high‐grade intraepithelial neoplasia, as determined by endoscopic diagnosis. Patients with a history of chemotherapy, radiotherapy, chemoradiotherapy, or surgery for ESCC were excluded (Figure 1).

### Data preparation and annotation of lesion frames

**FIGURE 2:**
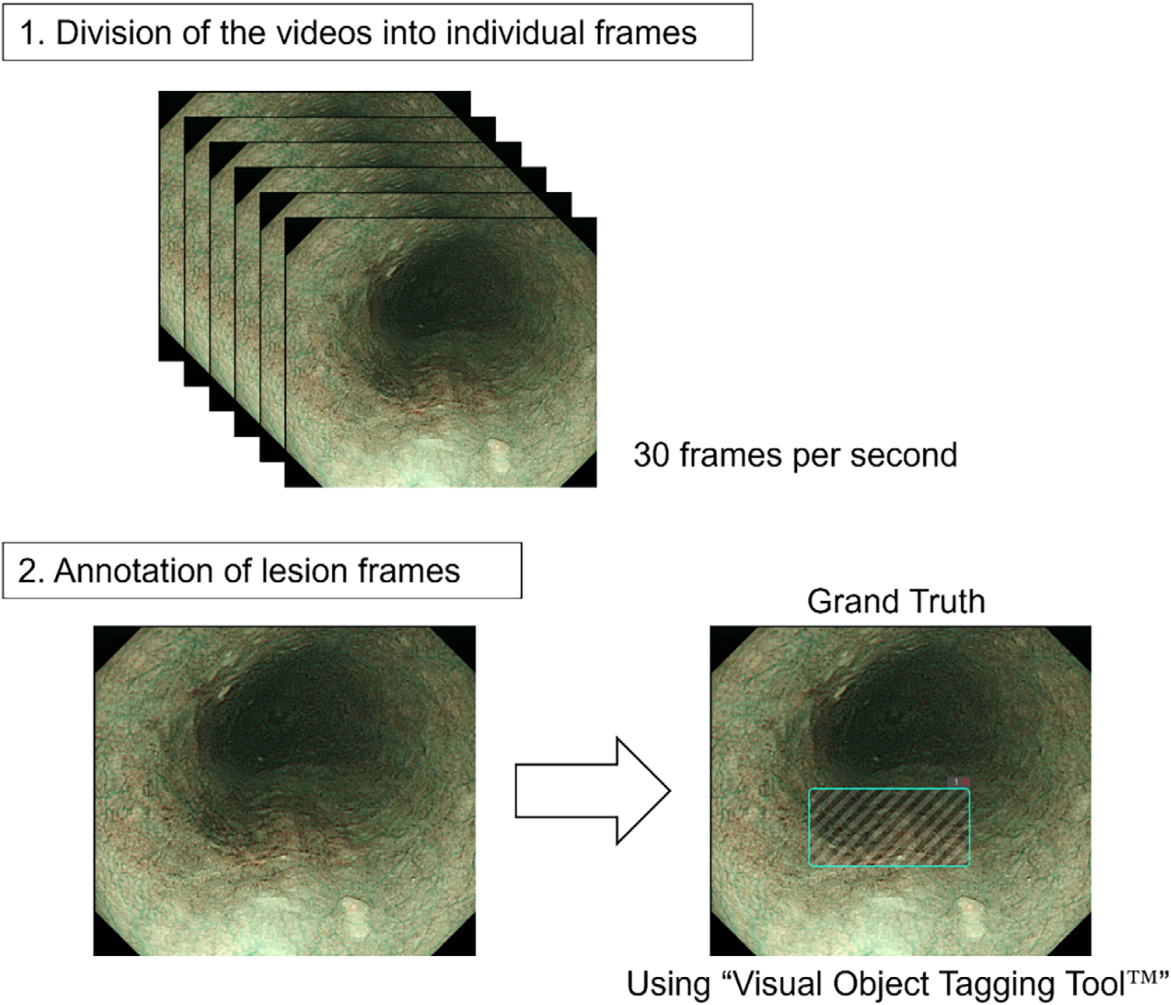
Overview of the data preparation and annotation process for lesion frames. 1. Videos were divided into individual frames of 30 fps each. 2. Lesion frames were annotated using the Visual Object Tagging Tool to create a ground‐truth dataset.

Videos depicting mucosa with or without neoplastic lesions were divided into individual frames for subsequent analysis. Within this dataset, the quality of certain frames was suboptimal, characterized by image blurring and artifacts, such as bubbles, to the extent that lesion identification remained feasible. Frames of the ESCC videos were annotated by four skilled endoscopists at the NCCHE using the Visual Object Tagging Tool developed by Microsoft Commercial Software Engineering, under the MIT license (https://github.com/microsoft/VoTT/blob/master/LICENSE). Each frame in the videos was assigned a rectangular box based on reference images, such as iodine‐stained images, obtained during pre‐endoscopic submucosal dissection (ESD) examination and from iodine‐stained ESD specimens. This annotation provided ground‐truth data for training and validating the AI model to differentiate videos with and without neoplastic lesions (Figure 2).

### AI model development

We utilized the You Only Look Once v3 machine‐learning model, a state‐of‐the‐art object‐detection algorithm.^22^ The model was trained using annotated ESCC and normal mucosa videos obtained from the NCCHE in order for it to learn the distinctive patterns and features associated with ESCC. Iterative optimization processes enhanced the model's performance and accuracy, including fine‐tuning the model parameters and adjusting the training process based on the validation results.

### Evaluation of the AI model

**FIGURE 3:**
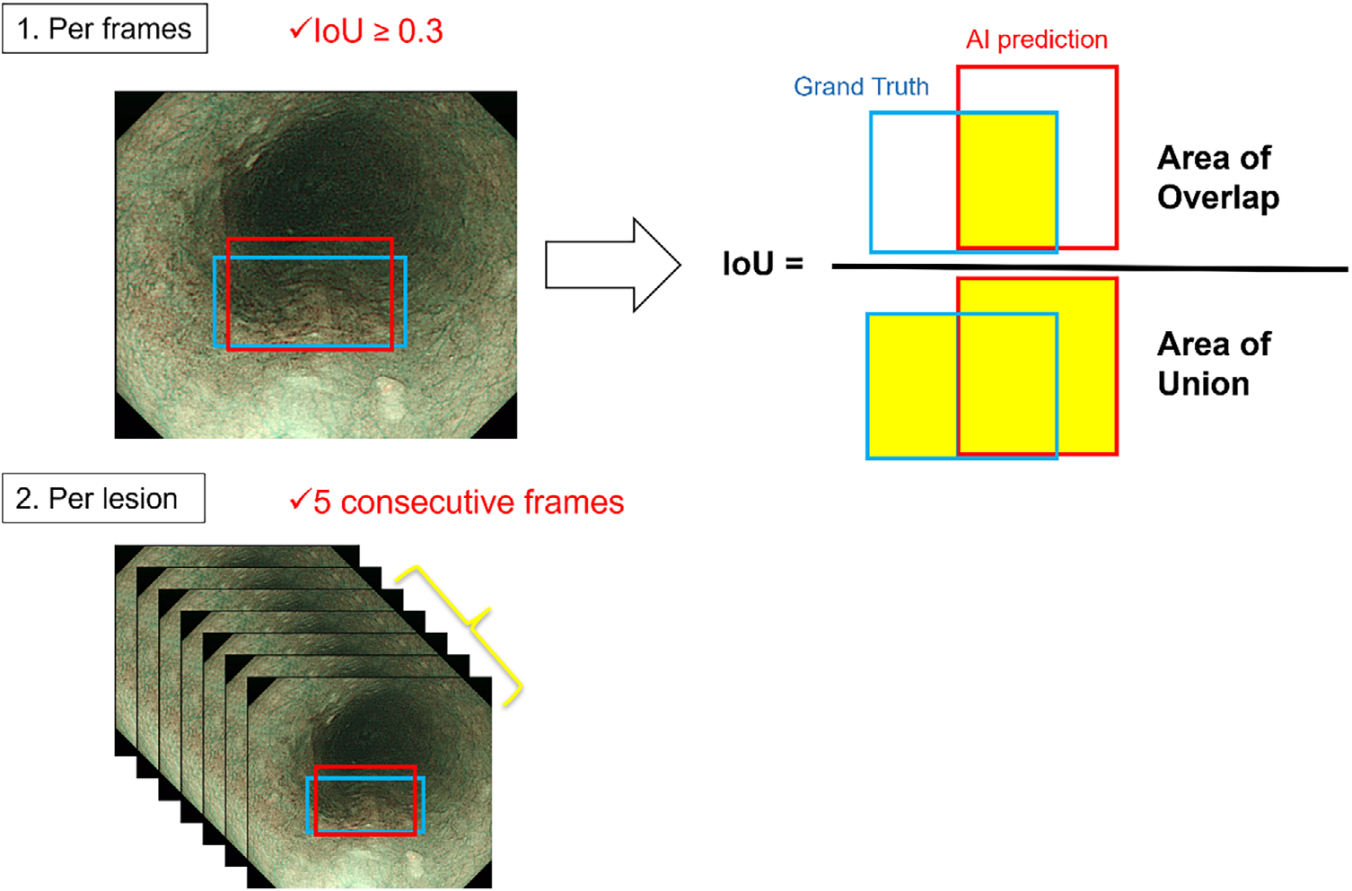
Definition of lesion detection using the AI model. 1. Per‐frame analysis involved evaluating the IoU with a threshold of ≥ 0.3 and comparing the ground truth and AI predictions for each frame. The IoU was calculated as the area of overlap divided by the area of union. 2. Per‐lesion analysis considered detection over five consecutive frames. AI, artificial intelligence; IoU, intersection over union.

Short video clips lasting 3–5 s were extracted from the captured videos for testing. An equal number of video clips depicting the lesion in either the close‐up or distant view were prepared. The distant view refers to a perspective captured from a far position at which abnormal blood vessels cannot be identified. In contrast, the close‐up view refers to a perspective captured from a closer position where abnormal blood vessels become visible. Importantly, in the close‐up view, we only employed low‐level magnification; no high‐level magnification was applied for detailed examination. The model's diagnostic capabilities were evaluated. In the per‐frame analysis, we defined the detection of lesions by the AI model with IoU (Intersection over Union) ≥0.3 as a true positive. In the per‐lesion analysis, a lesion that was correctly detected in five consecutive frames was defined as a true positive (Figure 3). Subsequently, the model's performance was evaluated by comparing its predictions with the diagnoses made by a group of endoscopists, including skilled experts and non‐experts. Eighteen endoscopists, comprising eight experts and ten non‐experts, were recruited from two institutions. The categorization of endoscopists as experts or non‐experts was determined by their certification status as board‐certified fellows of the Japan Gastroenterological Endoscopy Society. The endoscopists independently reviewed the test videos captured at the NCCHE and provided their diagnoses. The participants were tasked with determining only the presence or absence of lesions in the videos. Sensitivity, specificity, accuracy, positive predictive value, negative predictive value, and interobserver agreement were calculated to assess the diagnostic performance of both the AI model and endoscopists (Figure 1). This study was designed as a retrospective analysis.

### Assessment of AI assistance

To explore the model's assistance ability, endoscopists were asked to diagnose the test data again after a 4‐week washout interval while referring to the results predicted by the AI model. The diagnostic performance of the endoscopists with and without AI assistance was compared to assess the effects of AI assistance on their diagnostic accuracy (Figure 1).

### Statistical analysis

Descriptive analyses were conducted to summarize the diagnostic performance measures of the AI model and endoscopists. The comparison of diagnostic performance between the AI model and endoscopists was determined to yield a significant difference if the diagnostic performance of the AI was outside the 95% confidence interval of the mean diagnostic performance of the endoscopists. The diagnostic performances of AI‐assisted and non‐AI‐assisted endoscopists were compared using a paired t‐test and Wilcoxon matched‐pairs signed‐rank test. Additionally, the McNemar test was performed to assess whether AI assistance significantly improved the diagnostic performance of endoscopists. Subgroup analyses were performed based on lesion size, depth, location, endoscopic system, and other relevant factors. Statistical significance was set at p < 0.05. Interobserver agreement among the endoscopists was assessed using Fleiss' kappa (κ) coefficient. The criteria for the interpretation of kappa values by Landis and Koch were employed (poor: < 0.00, slight: 0.00–0.20, fair: 0.21–0.40, moderate: 0.41–0.60, substantial: 0.61–0.80, almost perfect agreement: >0.80). Statistical analyses were conducted using JMP Pro, version 17.1.0 (SAS Institute Inc.,) and GraphPad Prism, version 10.0.2, for Windows (GraphPad Software, www.graphpad.com).

### Ethical considerations

This study complied with the ethical principles outlined in the Declaration of Helsinki. This study involved the removal of the patients' personal information and was approved by the Institutional Review Board of the National Cancer Center East (2022‐162). Informed consent was obtained from all the patients whose videos were included in the study.

## RESULTS

### Patient and lesion characteristics

The data of 280 patients, including 140 with lesions and 140 without lesions, were included as training data. An additional 115 patients, comprising 52 with lesions and 63 without lesions, were used as test data. The clinicopathological characteristics of the test dataset lesions revealed a median tumor diameter of 14.5 mm (range: 1–100 mm). Regarding the invasion depth of the lesions, the majority were limited to the epithelium (*n* = 21), followed by the lamina propria mucosae (*n* = 17) and muscularis mucosae (n = 7). Statistical differences between the training and test data were evaluated using the Wilcoxon rank‐sum test for continuous variables and Fisher's exact test for categorical variables (Table 1).

**TABLE 1:** Patient and lesion characteristics in the training and test datasets.

	Training dataset	Test dataset	
Patient characteristics	*n* = 122	*n* = 47	*p*‐value
Sex: (male/female)	103 / 19	39 / 8	*p* = 0.818
Median age: year (range)	71 (37–91)	73 (49–87)	*p* = 0.436

Lesion characteristics	*n* = 140	*n* = 52	
Median tumor size: mm (range)	16 (1–107)	14.5 (1–100)	*p* = 0.938
Size: 1–10 mm / 11–20 mm / ≥21 mm	36 / 54 / 50	17 / 13 / 22	*p* = 0.210
Macroscopic types: 0‐IIa / 0‐IIb / 0‐IIc	3 / 4 / 133	1 / 1 / 50	*p* = 1.00
Location: Ce / Ut / Mt / Lt / Ae	1 / 30 / 74 / 35 / 0	0 / 5 / 32 / 15 / 0	*p* = 0.234
Aw / Pw / Rw / Lw	29 / 34 / 46 / 31	7 / 18 / 19 / 8	*p* = 0.332
Depth: HGIN / EP / LPM / MM / SM1 / SM2	0 / 74 / 43 / 14 / 4 / 5	3 / 21 / 17 / 7 / 0 / 4	*p* = 0.0392
Histological types: HGIN / SCC / others	0 / 140 / 0	3 / 49 / 0	*p* = 0.0190
Gastroscope: H260Z / H290Z / EZ1500 / XZ1200 / others	80 / 1 / 27 / 31 / 1	0 / 0 / 0 / 50 / 2	*p* <0.0001
Endoscopy system: CV‐260 / CV‐290 / CV‐1500	1 / 17 / 122	0 / 19 / 33	*p* = 0.0004

Abbreviations: Ae, abdominal esophagus; Aw, anterior wall; Ce, cervical esophagus; EP, epithelial; HGIN, high‐grade intraepithelial neoplasia; LPM, lamina propria mucosae; Lt, lower thoracic esophagus; Lw, left wall; MM, muscularis mucosae; Mt, middle thoracic esophagus; Pw, posterior wall; Rw, right wall; SCC, squamous cell carcinoma; SM, submucosa; Ut, upper thoracic esophagus.

### Evaluation of AI performance in isolation

The model's performance in detecting superficial ESCC was evaluated using the test data based on endoscopic video analysis. The AI model exhibited a sensitivity, specificity, and accuracy of 76.0%, 79.4%, and 77.2%, respectively.

### Assessment of the supportive effects of AI

**FIGURE 4:**
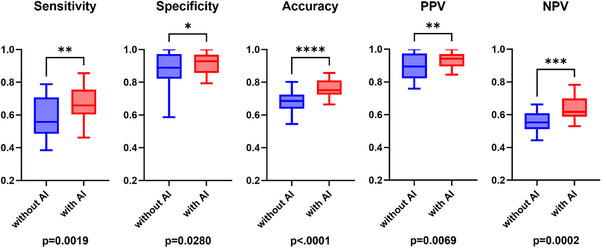
Diagnostic performance of all endoscopists with and without AI assistance. Significant improvements were observed with AI support across all evaluation criteria; particularly, a substantial enhancement in sensitivity and accuracy was noted. AI: artificial intelligence, PPV: positive predictive value, NPV: negative predictive value.

The effects of AI assistance on the diagnostic performance of endoscopists were assessed. Without AI assistance, the endoscopists demonstrated a sensitivity of 57.4%, a specificity of 87.0%, and an accuracy of 68.6%. However, AI assistance significantly improved the sensitivity (66.5%, *p* = 0.0019), specificity (91.6%, *p* = 0.0280), and accuracy (75.9%, *p* < 0.0001) of the endoscopists (Table 2 and Figure 4). The McNemar test further confirmed significant overall improvement with AI assistance (χ^2^ = 74.34, *p* < 0.0001). Interobserver agreement among the endoscopists was assessed with and without AI assistance. The Fleiss' kappa statistic (κ) showed an improvement from 0.429 (without AI assistance) to 0.547 (with AI assistance).

**TABLE 2:** Diagnostic performance of the artificial intelligence (model and endoscopists.

	Sensitivity, % (95% CI)	Specificity, % (95% CI)	Accuracy, % (95% CI)	PPV, % (95% CI)	NPV, % (95% CI)
**AI model**	76.0	79.4	77.2		
**Expert** (**n** = 8)					
Without AI	59.1 (51.9–66.3)	93.5 (88.8–98.1)	72.1 (67.9–76.3)	94.0 (90.3–97.7)	58.5 (54.2‐62.8)
With AI	70.0 (60.8–79.1)^*^	94.6 (91.0–98.3)	79.3 (74.5–84.1)^*^	95.9 (93.5–98.3)	66.6 (60.2–73.0)^*^
**Non‐expert** (**n **= 10)					
Without AI	56.1 (45.1–67.0)	81.9 (71.9–91.9)	65.8 (61.9–69.7)	85.6 (79.5–91.7)	54.3 (49.9–58.7)
With AI	63.7 (57.1–70.2)^*^	89.2 (84.2–94.2)^*^	73.3 (70.2–76.4)^*^	91.3 (87.6–95.0)^*^	60.4 (56.5–64.2)^*^
**All** (**n** = 18)					
Without AI	57.4 (51.2–63.6)	87.0 (80.9–93.1)	68.6 (65.6–71.6)	89.3 (85.3–93.3)	56.1 (53.2–59.1)
With AI	66.5 (61.4–71.5)^*^	91.6 (88.4–94.8)^*^	75.9 (73.1–78.8)^*^	93.4 (91.0–95.7)^*^	63.1 (59.6–66.6)^*^

Abbreviations: AI, artificial intelligence; NPV, negative predictive value; PPV, positive predictive value.

* *p* < 0.05, vs. without AI.

### Subgroup analysis

**FIGURE 5:**
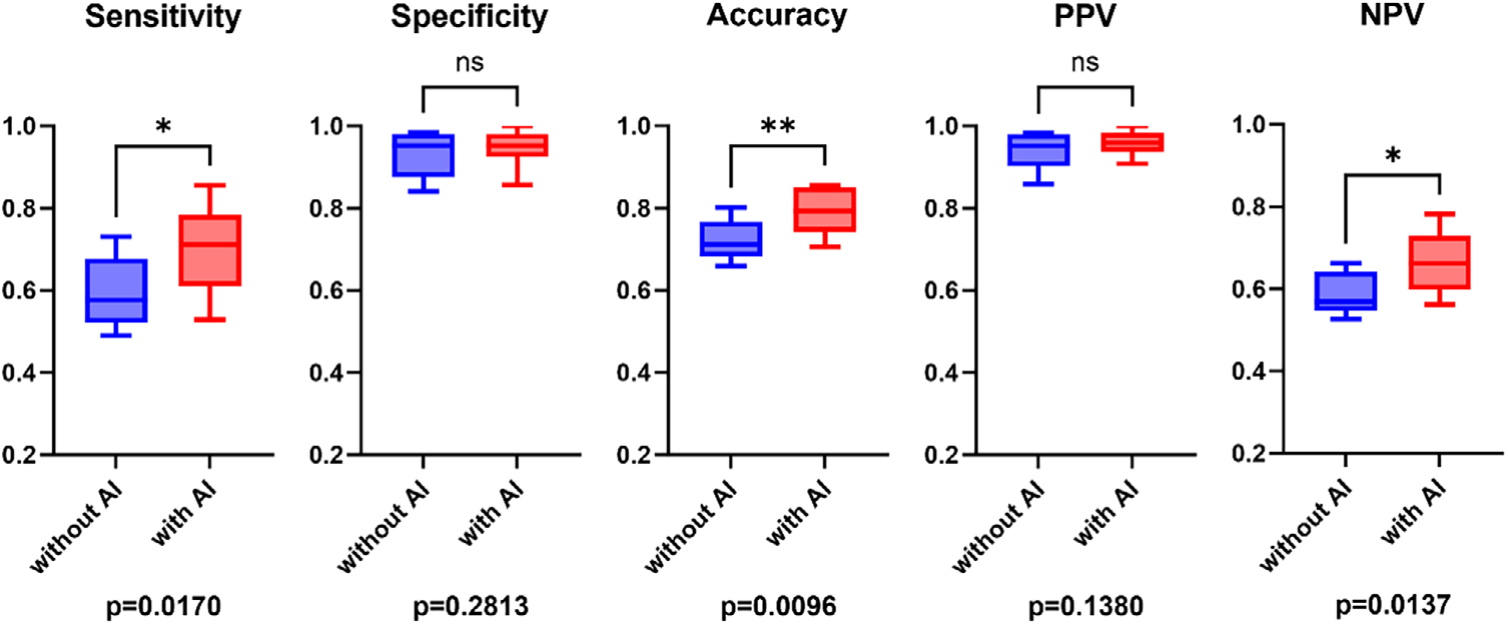
Diagnostic performance of expert endoscopists with and without AI assistance. Although there was a marginal improvement in the initially high sensitivity and PPV, statistical significance was not achieved. Notably, substantial enhancements were observed in sensitivity and accuracy. AI, artificial intelligence; PPV, positive predictive value; NPV, negative predictive value.

**FIGURE 6:**
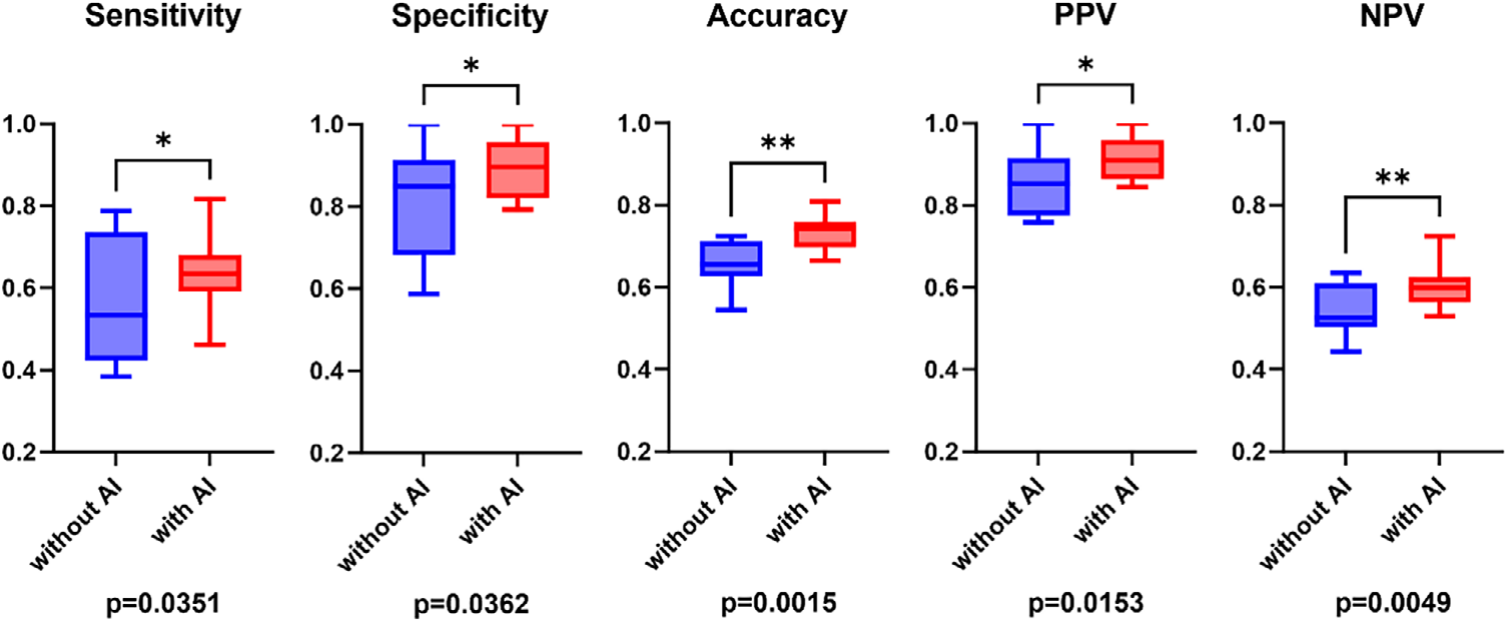
Diagnostic performance of non‐expert endoscopists with and without AI assistance. Significant improvements with AI support were observed across all evaluation criteria. A substantial improvement in specificity, sensitivity, and accuracy was a distinctive finding. AI, artificial intelligence; PPV, positive predictive value; NPV, negative predictive value.

**FIGURE 7:**
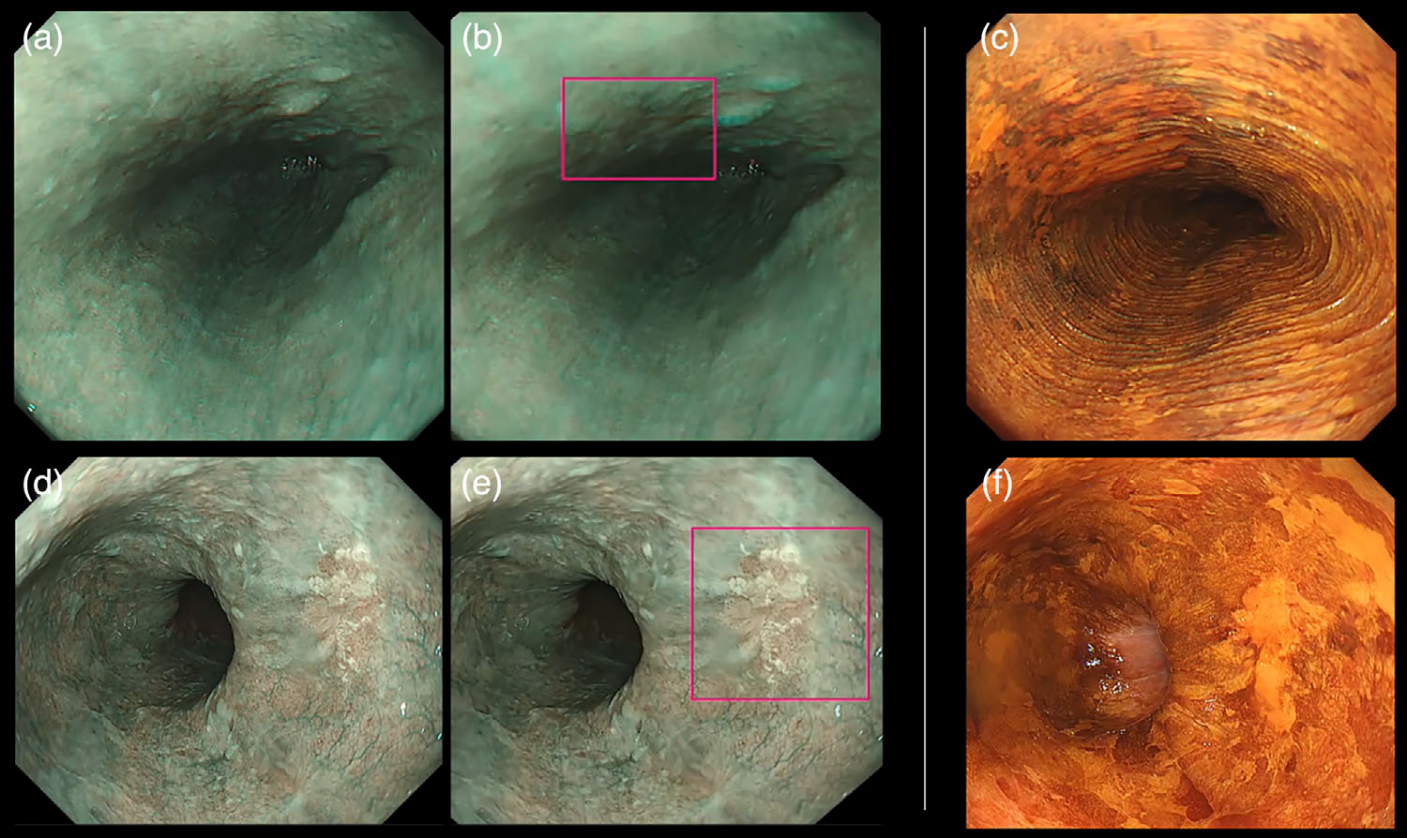
Examples of video‐captured images diagnosed using the AI model Images (a) and (d) depict instances of diagnosis without AI support, whereas images (b) and (e) represent cases diagnosed with AI assistance. The AI model accurately identified subtle changes in lesions in both distant and close‐up views. Images (c) and (f) served as the reference iodine‐stained images used in the creation of the ground truth. AI, artificial intelligence.

On analyzing the performances of experts and non‐experts separately, similar trends were observed. Among the experts, sensitivity and accuracy significantly improved with AI assistance (59.1% vs. 70.0%, *p* = 0.0170 and 72.1% vs. 79.3%, *p* = 0.0096, respectively; Table 2 and Figure 5). In the case of non‐experts as well, there was a significant improvement in sensitivity (56.1% vs. 63.7%, *p* = 0.0351), specificity (81.9% vs. 89.2%, *p* = 0.0362), and accuracy (65.8% vs. 73.3%, *p* = 0.0015; Table 2 and Figure 6). The analysis also differentiated between two distinct perspectives: the distant and close‐up views. Overall, the results were less favorable in the distant view context (Figure S1). Importantly, a consistent enhancement in performance was evident across both distant and close‐up perspectives when AI was integrated. Specifically, in the case of the distant view, there was a notable improvement in sensitivity (51.0%–59.8%, *p* = 0.0114), specificity (87.0%–91.6%, *p* = 0.0295), and accuracy (70.7%–77.2%, *p* < 0.0001; Figures S2 and S3). These improvements were consistently observed across different lesion sizes. Even in the case of small lesions (tumor size: 1–10 mm), significant enhancements in sensitivity (54.1% to 65.4%, *p* = 0.0070), specificity (87.0%–91.6%, *p* = 0.0280), and accuracy (75.5%–82.4%, *p* = 0.0001) were noted. The incorporation of AI resulted in a consistent cumulative benefit irrespective of lesion size (Figure S4). Furthermore, upon examining differences in each evaluation criterion between facilities, no significant difference was observed in any of the criteria (Figure S5). Examples of video‐captured images diagnosed by the AI model are shown in Figure 7.

## DISCUSSION

In this study, we developed an AI model to assist in the detection and diagnosis of superficial ESCC using endoscopic video analysis. The model's performance was promising, with a sensitivity of 76.0%, specificity of 79.4%, and accuracy of 77.2%. The AI model exhibited higher sensitivity and accuracy than endoscopists alone. Moreover, the diagnostic support provided by the AI to the endoscopists was demonstrated across all the evaluated criteria. This observation was particularly notable in areas where the baseline performance was relatively suboptimal; thus, room for improvement was more substantial. These results highlight the potential of AI technology to support endoscopists in detecting and diagnosing esophageal lesions in a clinical setting.

Several studies have examined the effects of augmenting the ESCC diagnostic capabilities of endoscopists using AI.^16^, ^17^, ^18^, ^23^, ^24^, ^25^ Both previous studies and the current study have recognized the added advantages of AI assistance for endoscopists. Previous studies on the added benefits of AI assistance for endoscopists have predominantly relied on still images for testing, with limited validation using video‐based assessments.^17^, ^18^ Furthermore, most studies were focused on white light imaging, with few investigations using narrow‐band imaging.^17^, ^24^ While two RCTs have been published,^26^, ^27^ there are some points for discussion, such as the inclusion of lesions detectable only by iodine staining or those identified after iodine chromoendoscopy, as well as how low‐grade intraepithelial neoplasia is classified. Our study explicitly excludes these lesions, making it more aligned with real‐world clinical practice.

Our study focused on evaluating the effects of AI assistance on the diagnostic performance of both experts and non‐experts. The findings revealed that AI assistance significantly improved the diagnostic performance of both skilled and novice endoscopists. This suggests that AI technology can be beneficial across different levels of expertise, supporting endoscopists in their clinical decision‐making.

A more detailed analysis of the results revealed that AI assistance resulted in a more significant improvement in the sensitivity of experts and specificity of non‐experts. It has been postulated that both experts and non‐experts tend to prioritize specificity over sensitivity in endoscopic diagnosis, possibly because training focuses on distinguishing between malignant and benign lesions rather than on identifying the lesion itself. With experience, there has been a shift in focus towards improved sensitivity. In our study, without AI assistance, experts and non‐experts differed in specificity rather than in sensitivity. AI helps experts recognize subtle lesions, thereby enhancing sensitivity while maintaining specificity. For non‐experts, AI guidance strengthens judgment capabilities, thereby improving specificity. Prioritizing sensitivity in the AI design may balance this inclination towards specificity among endoscopists.

Our study found that AI assistance was valuable across different lesion sizes, suggesting the model's versatility. We also compared distant and close‐up lesion observations using this novel approach. Close‐up observation plays a critical role in detecting fine details, such as abnormal blood vessels, which are difficult for AI to identify in distant views. Conversely, distant‐view observation presents challenges, including difficulties in detecting small or poorly defined lesions and insufficient lighting. Enhancing AI capabilities for distant‐view lesion detection could address these limitations and further improve diagnostic accuracy, especially in challenging scenarios.

To minimize selection bias, cases were mechanically classified based on the collection period to establish the training and validation datasets. Consequently, although unintentionally, the validation set primarily comprised videos captured using Olympus's latest equipment (GIF‐XZ1200), whereas the majority of the training set comprised videos captured with an older‐generation scope (GIF‐H260Z).

Generally, high‐resolution endoscopic images are presumed to enhance gastrointestinal lesion detection.^28^, ^29^, ^30^, ^31^, ^32^ Previous studies comparing AI performance between scopes with low (GIF‐Q260) and high resolution (GIF‐H260 and H290) did not observe any significant differences.^16^ While a discrepancy in the scope specifications between the training and validation datasets was encountered in this study, indicating a potential disadvantage for AI due to being insufficiently trained on high‐resolution lesion videos, the results still suggested a positive augmentation effect on the performance of endoscopists, similar to the findings of previous reports. This indicates the applicability of information obtained from older generation scopes relative to that obtained using the latest equipment, even if optical advancements continue. Although the continuous updating of AI models is deemed essential, the ability to utilize older data during updates is crucial.

Although there are benefits of using AI, caution is necessary when using it. The integration of AI as an assistive tool should be approached with care, ensuring that endoscopists possess fundamental endoscopic and critical thinking skills. Interpreting the AI results within the clinical context of patients is essential. These considerations emphasize the need for continuous research and the careful application of AI in clinical practice.

Although our study produced noteworthy results, it is essential to acknowledge its limitations. The foremost is the lack of a real‐time diagnostic evaluation in an actual clinical setting, which represents a significant constraint in assessing the practical effectiveness of our AI system.

Additionally, the reliance on data from a single medical facility to develop an AI model presents another limitation. Despite this limitation, a significant aspect of our research was the examination of the effects of AI assistance on the performance of endoscopists from various facilities. Recognizing the possibility of selection bias, it is crucial that endoscopists from diverse facilities beyond the collection site (NCCHE), including Kyoto University, participate in the trial. This inclusion of additional facilities is pivotal for improving the model's generalizability.

Furthermore, it is vital to emphasize that our study consistently demonstrated the positive effects of AI on all evaluation criteria across all facilities. Importantly, when potential differences in each evaluation criterion between facilities were closely examined, no significant variations were found. This result strongly indicates the model's versatility. To further ensure its generalizability, accumulating diverse cases from multiple facilities will be essential in future studies.

In conclusion, our study demonstrated the potential of AI technology to assist endoscopists in detecting and diagnosing superficial ESCC. The performance of the developed AI model in accurately identifying suspicious lesions was favorable, and its integration significantly improved the diagnostic capabilities of endoscopists. These findings have important implications for clinicians, as AI assistance can enhance the accuracy and consistency of the detection of early‐stage esophageal cancer, potentially leading to earlier diagnosis and improved patient outcomes. Further research is warranted to validate and refine the AI model, explore its utility in real‐world clinical practice, and address the limitations of this study.

## CONFLICT OF INTEREST STATEMENT

Tomonori Yano received financial support for the research from Olympus Medical Systems Corporation.

## ETHICS STATEMENT

Approval of the research protocol by an Institutional Reviewer Board: This study was approved by the Institutional Review Board of the National Cancer Center East (2022‐162).

## PATIENT CONSENT STATEMENT

Informed consent was obtained from all the patients whose videos were included in the study.

## CLINICAL TRIAL REGISTRATION

N/A

## Supporting information




**FIGURE S1** Comparison of diagnostic performance between distant and close‐up views. Results were less favorable in the distant view context.
**FIGURE S2** Diagnostic performance in the close‐up view with and without AI assistance. A consistent enhancement in diagnostic performance was evident in the close‐up perspectives when AI was integrated. AI: artificial intelligence.
**FIGURE S3** Diagnostic performance in the distant view with and without AI assistance. A consistent improvement in diagnostic performance was observed with the integration of AI support both in distant and close‐up views. AI: artificial intelligence.
**FIGURE S4** Diagnostic performance according to tumor size (A. 1–10 mm, B. 11–20 mm, C. ≥21 mm) with and without AI assistance. Consistent improvement in diagnostic performance across diverse lesion sizes was observed with the incorporation of AI. Even small lesions showed significant enhancement. AI: artificial intelligence.
**FIGURE S5** Comparison of diagnostic performance between NCCHE and Kyoto University. No statistically significant differences were observed across facilities for any of the evaluation criteria. NCCHE: National Cancer Center Hospital East.
