# Supplementary material for: Effects of artificial intelligence assistance on endoscopist performance: Comparison of diagnostic performance in superficial esophageal squamous cell carcinoma detection using video‐based models
Source: DEN Open. 2025 May 2;6(1):e70083. doi: 10.1002/deo2.70083 (PMC12046500; doi:10.1002/deo2.70083)

Supplementary Figure 1.


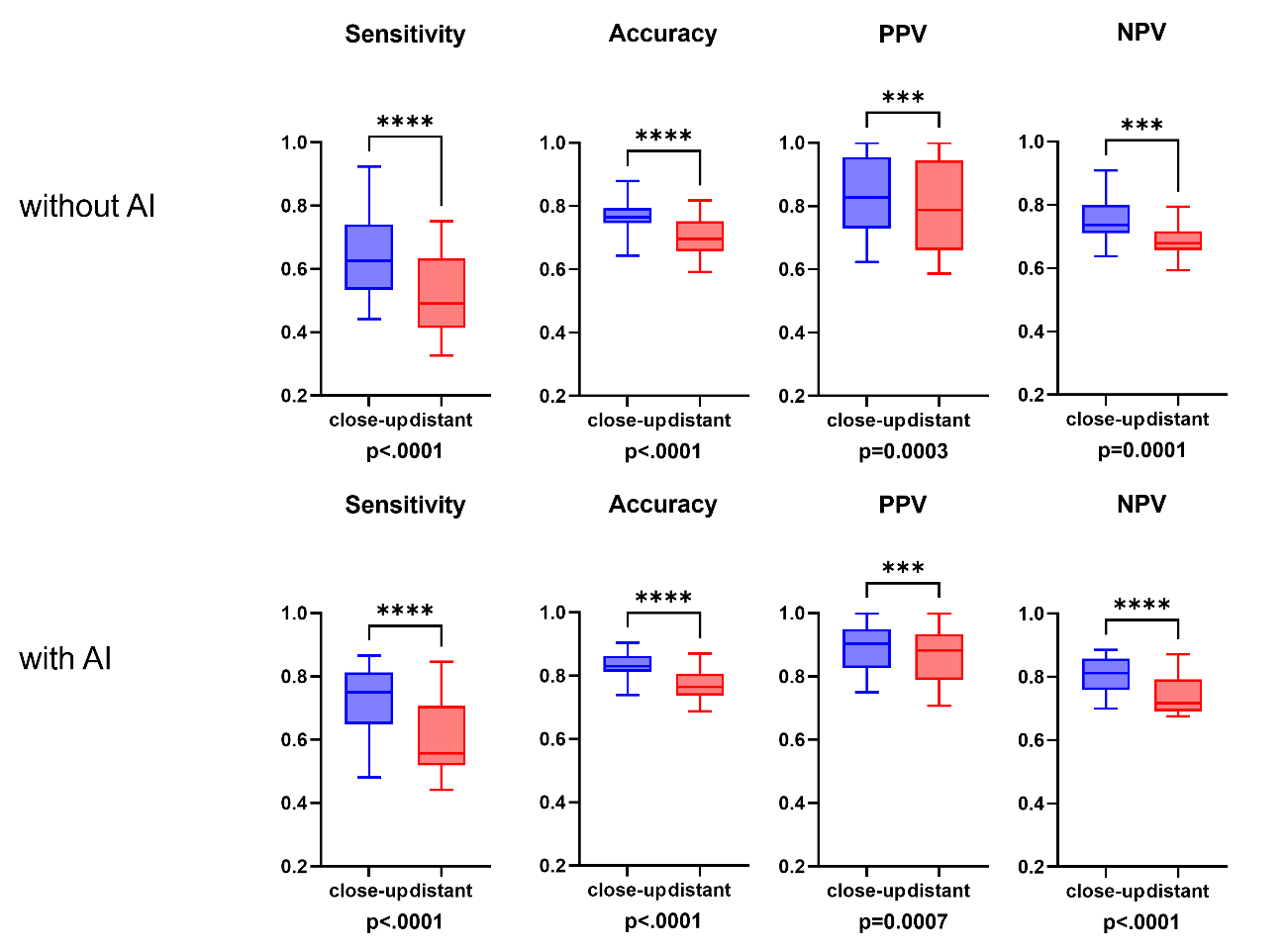


Supplementary Figure 2.


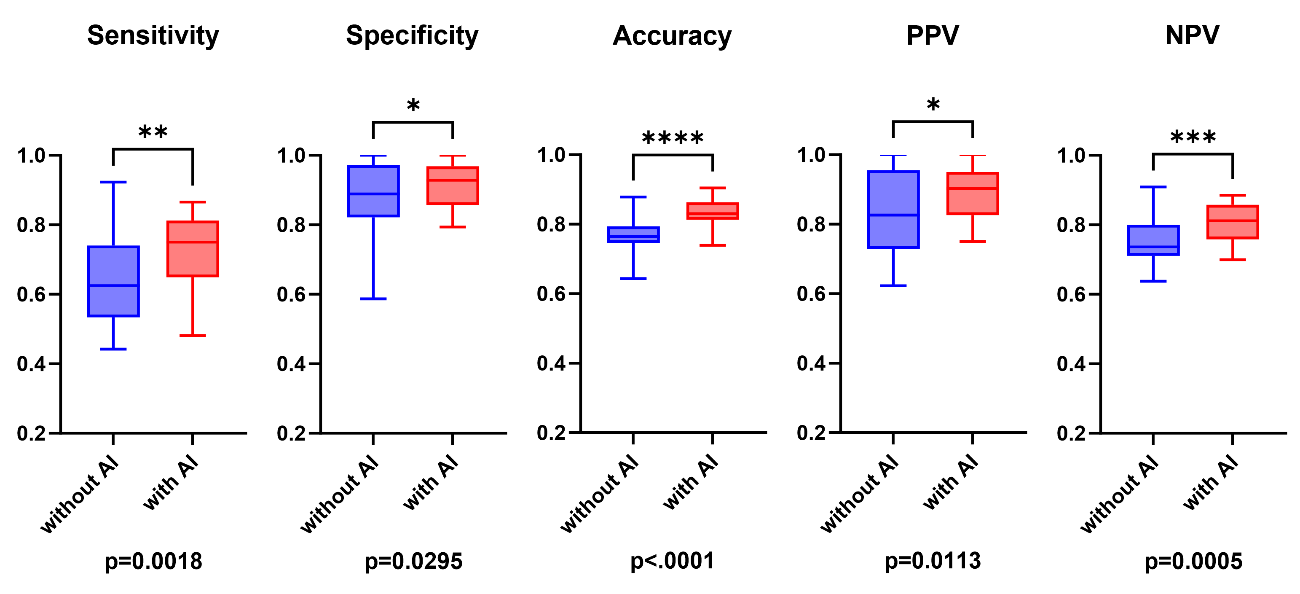


Supplementary Figure 3.


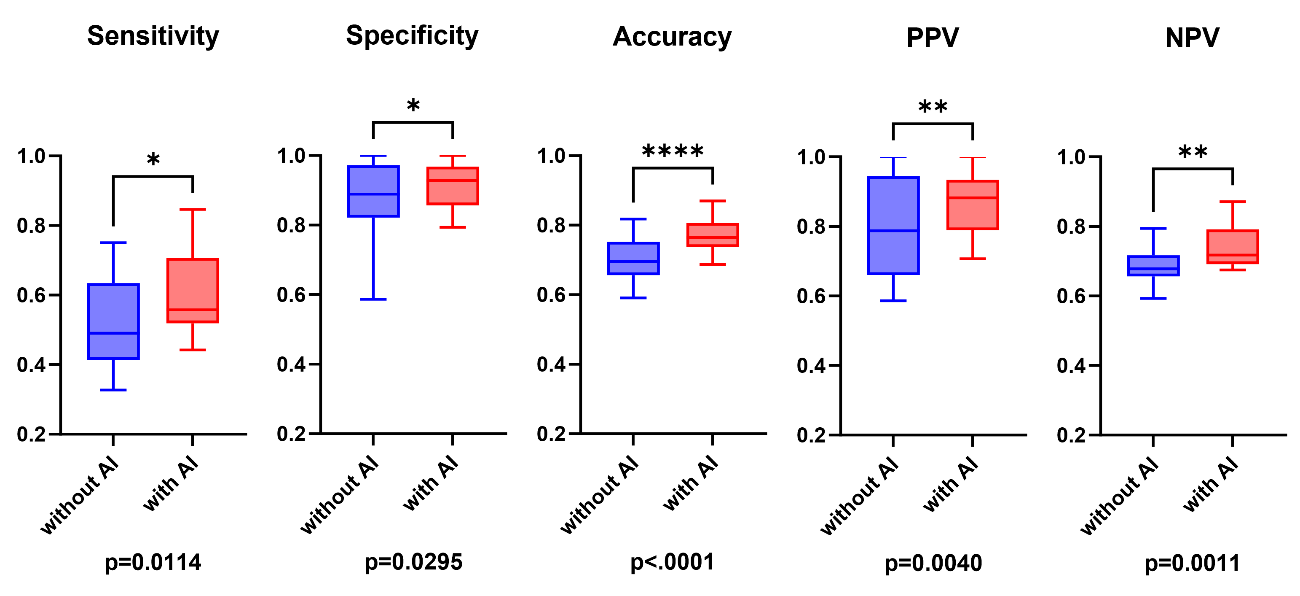


Supplementary Figure 4.


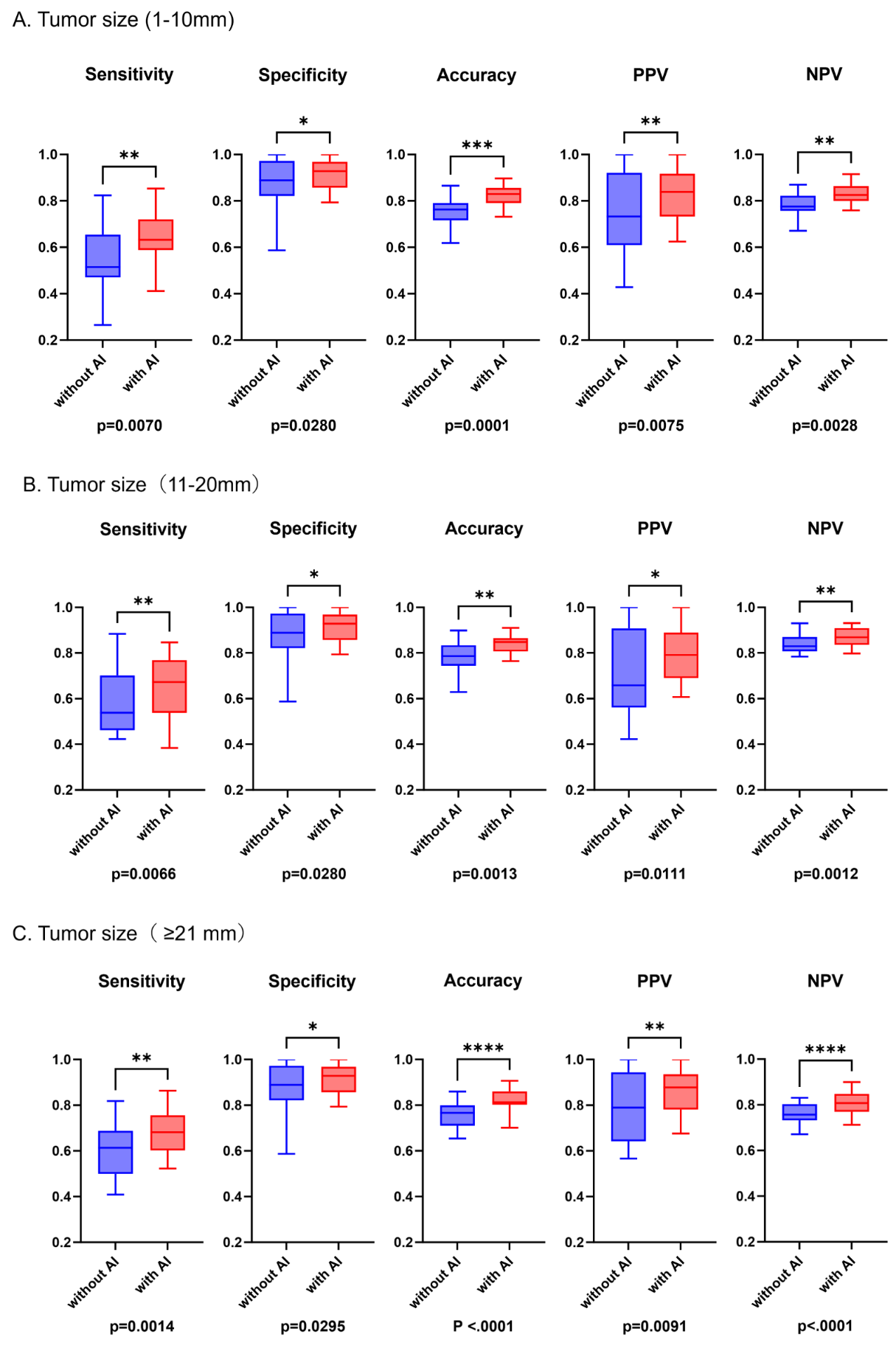


Supplementary Figure 5.


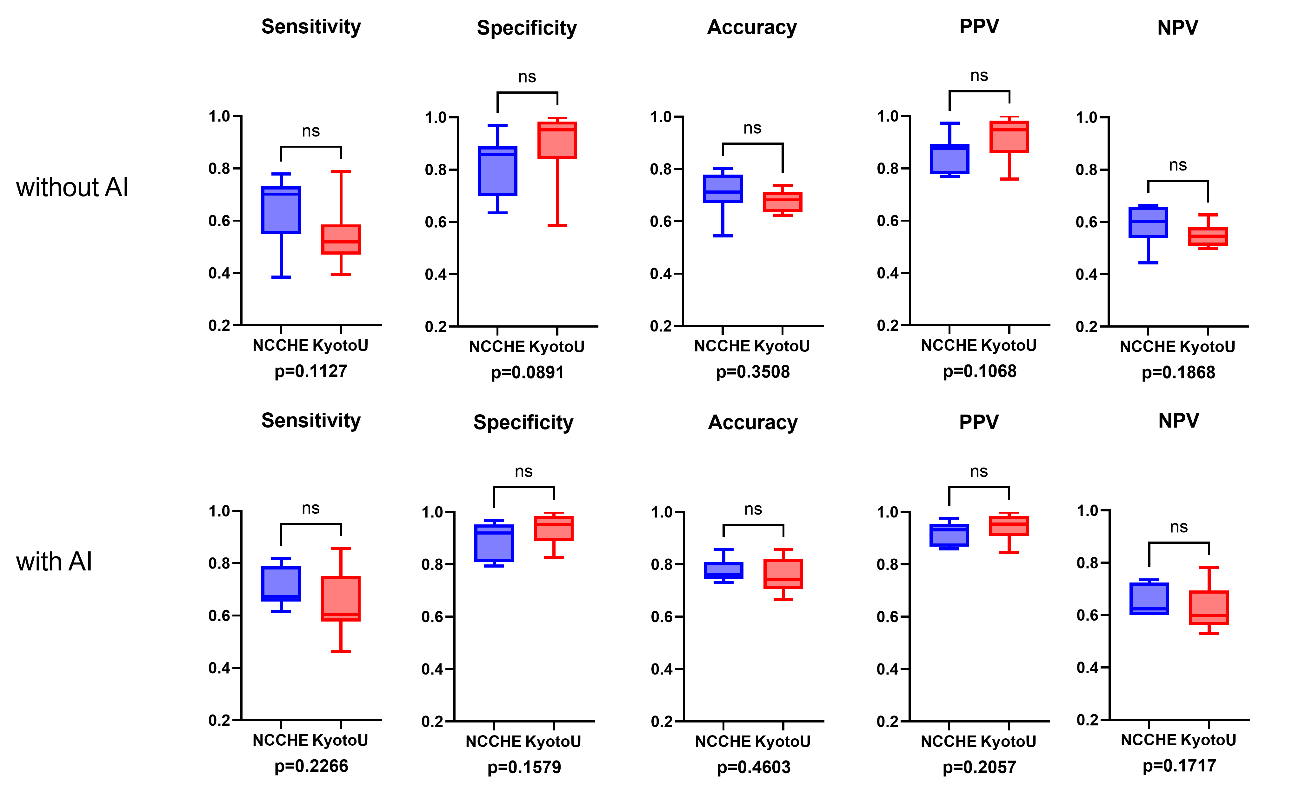

Supplement: Supplementary file 1 — FIGURE S1 Comparison of diagnostic performance between distant and close‐up views. Results were less favorable in the distant view context. FIGURE S2 Diagnostic performance in the close‐up view with and without AI assistance. A consistent enhancement in diagnostic performance was evident in the close‐up perspectives when AI was integrated. AI: artificial intelligence. FIGURE S3 Diagnostic performance in the distant view with and without AI assistance. A consistent improvement in diagnostic performance was observed with the integration of AI support both in distant and close‐up views. AI: artificial intelligence. FIGURE S4 Diagnostic performance according to tumor size (A. 1–10 mm, B. 11–20 mm, C. ≥21 mm) with and without AI assistance. Consistent improvement in diagnostic performance across diverse lesion sizes was observed with the incorporation of AI. Even small lesions showed significant enhancement. AI: artificial intelligence. FIGURE S5 Comparison of diagnostic performance between NCCHE and Kyoto University. No statistically significant differences were observed across facilities for any of the evaluation criteria. NCCHE: National Cancer Center Hospital East. [file DEO2-6-e70083-s001.docx]
